# Supplementary material for: End Sequence Analysis Toolkit (ESAT) expands the extractable information from single-cell RNA-seq data
Source: Genome Res. 2016 Oct;26(10):1397–410. doi: 10.1101/gr.207902.116 (PMC5052061; doi:10.1101/gr.207902.116)
Supplement: Supplemental Material [file supp_gr.207902.116_Supp_Materials.tar.gz › ESAT-esat_v0.1/doc/html/class_jama_1_1_l_u_decomposition.html]

ESAT: Jama.LUDecomposition Class Reference


ESAT


- Main Page
- Related Pages
- Packages
- Classes
- Files

- Class List
- Class Index
- Class Hierarchy
- Class Members

All Classes Namespaces Files Functions Variables Enumerator Pages

- Jama
- LUDecomposition

Public Member Functions |
List of all members

Jama.LUDecomposition Class Reference

Inheritance diagram for Jama.LUDecomposition:

[legend]

Collaboration diagram for Jama.LUDecomposition:

[legend]

|  |  |
| --- | --- |
| Public Member Functions | |
|  | LUDecomposition (Matrix A) |
|  | |
| boolean | isNonsingular () |
|  | |
| Matrix | getL () |
|  | |
| Matrix | getU () |
|  | |
| int[] | getPivot () |
|  | |
| double[] | getDoublePivot () |
|  | |
| double | det () |
|  | |
| Matrix | solve (Matrix B) |
|  | |

## Detailed Description

LU Decomposition.

For an m-by-n matrix A with m >= n, the LU decomposition is an m-by-n unit lower triangular matrix L, an n-by-n upper triangular matrix U, and a permutation vector piv of length m so that A(piv,:) = L\*U. If m < n, then L is m-by-m and U is m-by-n.

The LU decompostion with pivoting always exists, even if the matrix is singular, so the constructor will never fail. The primary use of the LU decomposition is in the solution of square systems of simultaneous linear equations. This will fail if isNonsingular() returns false.

## Constructor & Destructor Documentation

|  |  |  |  |  |  |
| --- | --- | --- | --- | --- | --- |
| Jama.LUDecomposition.LUDecomposition | ( | Matrix | *A* | ) |  |

LU Decomposition

Parameters
:   |  |  |
    | --- | --- |
    | A | Rectangular matrix |

Returns
:   Structure to access L, U and piv.

## Member Function Documentation

|  |  |  |  |  |
| --- | --- | --- | --- | --- |
| double Jama.LUDecomposition.det | ( |  | ) |  |

Determinant

Returns
:   det(A)

Exceptions
:   |  |  |
    | --- | --- |
    | IllegalArgumentException | Matrix must be square |

|  |  |  |  |  |
| --- | --- | --- | --- | --- |
| double [] Jama.LUDecomposition.getDoublePivot | ( |  | ) |  |

Return pivot permutation vector as a one-dimensional double array

Returns
:   (double) piv

|  |  |  |  |  |
| --- | --- | --- | --- | --- |
| Matrix Jama.LUDecomposition.getL | ( |  | ) |  |

Return lower triangular factor

Returns
:   L

|  |  |  |  |  |
| --- | --- | --- | --- | --- |
| int [] Jama.LUDecomposition.getPivot | ( |  | ) |  |

Return pivot permutation vector

Returns
:   piv

|  |  |  |  |  |
| --- | --- | --- | --- | --- |
| Matrix Jama.LUDecomposition.getU | ( |  | ) |  |

Return upper triangular factor

Returns
:   U

|  |  |  |  |  |
| --- | --- | --- | --- | --- |
| boolean Jama.LUDecomposition.isNonsingular | ( |  | ) |  |

Is the matrix nonsingular?

Returns
:   true if U, and hence A, is nonsingular.

Here is the caller graph for this function:

|  |  |  |  |  |  |
| --- | --- | --- | --- | --- | --- |
| Matrix Jama.LUDecomposition.solve | ( | Matrix | *B* | ) |  |

Solve A\*X = B

Parameters
:   |  |  |
    | --- | --- |
    | B | A Matrix with as many rows as A and any number of columns. |

Returns
:   X so that L\*U\*X = B(piv,:)

Exceptions
:   |  |  |
    | --- | --- |
    | IllegalArgumentException | Matrix row dimensions must agree. |
    | RuntimeException | Matrix is singular. |

Here is the call graph for this function:

---

The documentation for this class was generated from the following file:

- java/Jama/LUDecomposition.java


---

Generated on Fri Aug 15 2014 15:11:06 for ESAT by  

 1.8.7
